# Family Structure and Its Changes and Depressive Symptoms in Later Life: How Intergenerational Support Makes a Difference

**DOI:** 10.3390/healthcare14131855

**Published:** 2026-06-25

**Authors:** Yaocheng Luo, Youtao Mou, Zhenzhen Peng, Peng Zeng, Lin Fu, Jiaxin Guo, Zumin Shi, Yong Zhao

**Affiliations:** 1 School of Public Health, Chongqing Medical University, Chongqing 400016, China; 2024111689@stu.cqmu.edu.cn (Y.L.); 2024121761@stu.cqmu.edu.cn (Y.M.); 2025121908@stu.cqmu.edu.cn (Z.P.); 2025121935@stu.cqmu.edu.cn (P.Z.); 2023121648@stu.cqmu.edu.cn (L.F.); 2023121654@stu.cqmu.edu.cn (J.G.); 2 Research Center for Medicine and Social Development, Chongqing Medical University, Chongqing 400016, China; 3 Research Center for Public Health Security, Chongqing Medical University, Chongqing 400016, China; 4 Nutrition Innovation Platform-Sichuan and Chongqing, School of Public Health, Chongqing Medical University, Chongqing 400016, China; 5 Human Nutrition Department, College of Health Sciences, QU Health, Qatar University, Doha 2713, Qatar; zumin@qu.edu.qa

**Keywords:** depressive symptoms, family structure, intergenerational support, middle-aged and older adults, CHARLS

## Abstract

Objectives: Depressive symptoms are common among middle-aged and older adults. This study examined the associations of family structure and its transitions with depressive symptoms, and the mediating role of intergenerational support. Methods: Participants were drawn from the first five waves of CHARLS. Family structure and transitions were assessed at baseline (Wave 1) and Wave 2. Outcome was defined as new-onset depressive symptoms occurring after Wave 2 among participants who were free of depressive symptoms at both Wave 1 and Wave 2. A Cox proportional hazards model was used to examine the association between family structure/transitions and incident depressive symptoms. Parallel mediation analysis was conducted to examine the potential mediating effects of intergenerational support. Results: Compared with two-generation households, skipped-generation households were associated with a higher risk of depressive symptoms in older adults (HR = 1.30, 95% CI: 1.04–1.63), and transitions from two-generation to skipped-generation households were also associated with a higher risk (HR = 1.54, 95% CI: 1.06–2.23). Emotional support partially mediated this association. Conclusions: Older adults living in or transitioning to skipped-generation households are associated with a higher risk of depressive symptoms, suggesting that public health efforts should prioritize skipped-generation households and the processes leading to their formation.

## 1. Introduction

China is undergoing a pronounced demographic aging crisis. In 2024, the population aged 65 years and older totaled 217 million, comprising 15.4% of the national population [1]. By 2031, China is expected to become a “super-aged” society, with the proportion of people aged 65 and older exceeding 20% [2]. Aging not only signifies an increased disease burden but also brings increasingly severe mental health challenges. As a widespread mental disorder defined by persistent low mood [3], depressive symptoms are highly prevalent in older populations. It is associated with cardiovascular diseases, dementia, and suicide in this demographic [4,5]. Evidence shows that 32.9% of middle-aged and older adults in China suffer from depressive symptoms, a rate considerably higher than the 3.4% prevalence observed among the general adult population [6]. The Healthy China 2030 Plan Outline has also incorporated the promotion of mental health as one of its key initiatives [7].

The family is the primary support system for middle-aged and older individuals, providing not only emotional comfort and daily care but also serving as a core component of the social support system [8]. Family structure refers to how family members are organized and interact within the family system. Stable and functionally intact family structures provide significant protection for the mental health of middle-aged and older individuals. However, with the implementation of the family planning policy, accelerated urbanization, and increased population mobility [9], the outmigration of young and middle-aged adults has led to a hollowing-out trend in rural areas, while the mobility of the elderly and children lags [10], leading to a gradual reduction in family size and the number of multigenerational households [11]. Findings from the Seventh National Population Census (2020) indicate that the mean household size fell from 3.10 individuals in 2010 to 2.62 individuals [12]. In contemporary China, intergenerational separation has emerged as a common and well-recognized aspect of family life [13], which may lead to changes in family structure types and a gradual reduction in intergenerational interactions among family members.

Family structure represents the external manifestation of intergenerational support, whereas intergenerational support constitutes the internal function of family structure [14]. Intergenerational support refers to the bidirectional exchange of material and emotional resources between parents and children [15], primarily encompassing emotional, financial, and caregiving support [16,17]. Typically, adult children provide financial assistance and emotional care to their parents, while older adults offer reciprocal support to younger generations, such as caring for grandchildren. Prior studies indicate that intergenerational support is related to the risk of depressive symptoms in middle-aged and older populations [18,19]. Intergenerational relationships within the family directly and positively influence the acquisition of intergenerational support [20]. Such support may mediate the association between various factors and depressive symptoms among middle-aged and older adults [21].

Several cross-sectional studies have explored the relationship between family structure and depressive symptoms [22,23], but the longitudinal evidence about the change in family structure is still relatively limited. In addition, the mediating role of intergenerational support in family structure change and depressive symptoms has rarely been systematically studied. The aim of this study is to explore the relationship between static family structures (two-generation, multigenerational, and skipped-generation households), dynamic family changes, and depressive symptoms in older adults, as well as the potential mediating role of intergenerational support in this process. Therefore, this study tested the following hypothesis: both static family structure and dynamic family changes can affect the incidence of depressive symptoms in the elderly, and intergenerational support may play a mediating role in this process.

## 2. Materials and Methods

### 2.1. Study Design and Population

Data were obtained from CHARLS, a nationally representative cohort of adults aged 45+ and their spouses. The survey aims to assess the impact of population aging on health and economic outcomes in China [24]. This study used 2011 baseline and 2013 follow-up data to examine changes in family structure, with participants subsequently tracked through 2020. CHARLS was approved by Peking University IRB (IRB00001052-11015), and informed consent was obtained from all participants. We applied to the CHARLS database online in January 2025 and were approved quickly.

**Figure 1:**
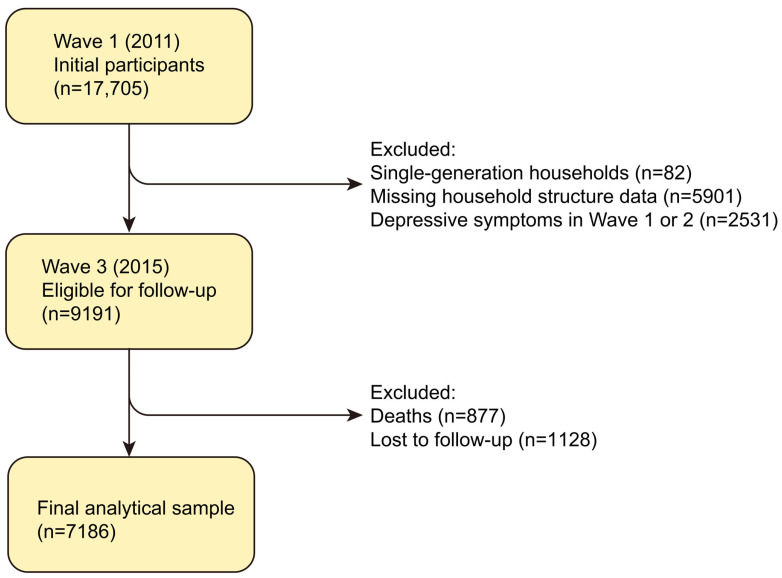
Selection process of the study population.

In accordance with the study objectives, participants were eligible if they were aged ≥45 years, had complete household composition information, and were free of depressive symptoms at baseline and the beginning of follow-up (2011 and 2013) to ensure temporal ordering between household structure and depressive symptoms (Figure 1). Among the 17,705 participants in the 2011 wave, 5983 individuals with one-generation households or missing household structure information were excluded. One-generation households were excluded due to the very small sample size (n = 82, 0.46%), while participants with missing household structure information could not be classified reliably. Subsequently, 2531 participants with depressive symptoms in 2011 or 2013 were excluded to focus on incident depressive symptoms. In addition, 2005 participants who died or were lost to follow-up were excluded because outcome status could not be ascertained. A total of 7186 participants were included in the final analysis.

### 2.2. Family Structure

This study classified family structure from an intergenerational perspective. Based on the CHARLS household questionnaire item “What is your relationship with a certain household member?”, family members were categorized into four generational levels: parental generation, same generation, child generation, and grandchild generation. Based on the number and types of generations within the household, family structures were categorized as one-generation, two-generation, multigenerational, and skipped-generation households [10]. As one-generation households included only 82 participants (0.46%), only two-generation, multigenerational, and skipped-generation households were included in the analysis to ensure model stability and facilitate interpretation of the results.

### 2.3. Outcome and Follow-Up

The primary outcome was depressive symptoms, assessed using the CES-D-10 in CHARLS. The CES-D-10 contains 10 items scored from 0 to 3, yielding a total score ranging from 0 to 30. Consistent with previous CHARLS-based studies, a CES-D-10 score of ≥20 was used in the primary analysis to identify participants with elevated depressive symptoms, representing a more conservative threshold for clinically meaningful depressive symptomatology [25]. In the primary analysis, participants who died or were lost to follow-up during the study period were excluded from the final analytical sample. Follow-up time was defined as the year of the first occurrence of depressive symptoms or the end of follow-up minus 2013. Incident depressive symptoms were defined as a binary outcome (1 = occurrence of depressive symptoms during follow-up, 0 = no occurrence of depressive symptoms during follow-up).

### 2.4. Intergenerational Support

In this study, intergenerational support was categorized into three dimensions—emotional support, financial support, and caregiving support—based on previous CHARLS-related studies [17]. Emotional support was operationalized as the frequency of intergenerational contact using two items from the 2013 CHARLS household questionnaire: “How often do you see your children?” and “How often do you contact your children via phone, mail, or text message?” The higher of the two item scores was used for analysis. Emotional support was measured by the frequency of emotional communication and interaction with family members. Higher scores indicate higher levels of emotional support. Financial support was measured by the item “Economic support received from children within the past year.” Caregiving support was measured by the item “How much time do you spend caring for your grandchildren?”

### 2.5. Covariates

The analysis controlled for covariates across three domains: sociodemographic characteristics, health status, and lifestyle factors. Sociodemographic characteristics included participants’ age, sex, educational attainment (illiterate, primary school, or high school or above), marital status (married or single), residence (urban or rural), and number of children. Health status variables included body mass index (BMI), self-rated health (poor, fair, or good), activities of daily living (ADL), instrumental activities of daily living (IADL), and non-communicable diseases (NCDs). Lifestyle variables encompassed sleep quality, smoking, and alcohol consumption.

### 2.6. Statistical Analysis

Descriptive analyses were first conducted to examine the sociodemographic characteristics and incidence of depressive symptoms across different family structures. Categorical variables were shown as counts and percentages, and continuous variables as means ± SD. Group differences were assessed using chi-square or *t*-tests. Cox models were used to assess the association between family structure and the incidence of depressive symptoms, with two-generation households as the reference group. Both Model 1 and Model 2 were constructed and tested using Schoenfeld residuals. Cox proportional hazards models were used to examine the association between family structure and incident depressive symptoms, with two-generation households as the reference group. The time origin for follow-up was defined as Wave 2. Participants were followed from Wave 2 until the first occurrence of depressive symptoms, death, loss to follow-up, or the end of the study period (censoring), whichever occurred first. Only the first occurrence of depressive symptoms was considered the outcome in the analysis, given repeated assessments across waves. The proportional hazards assumption was assessed using Schoenfeld residuals. A *p* value greater than 0.05 for both the primary exposure variable and the global test was considered to indicate that the proportional hazards assumption was satisfied.

Further analyses assessed the relationship between changes in family structure and the risk of depressive symptoms, with subgroup analyses conducted by sex, age, residence, and educational attainment. Finally, a parallel mediation model was employed using the regression-based approach implemented in the CMAverse package to examine the mediating role of intergenerational support. Family structure assessed at Wave 1 was treated as the exposure, intergenerational support measured at Wave 2 as the mediator, and incident depressive symptoms occurring after Wave 2 as the outcome. To account for the time-to-event nature of the outcome, a Cox proportional hazards model was specified for the outcome model, while linear regression models were used for the mediator models. All models were adjusted for demographic and health-related covariates. Natural direct effects, natural indirect effects, total effects, and the proportion mediated were estimated using 1000 bootstrap resamples to obtain 95% confidence intervals.

In addition, the following sensitivity analyses were conducted: (1) considering the competing risk between death and incident depressive symptoms, the primary analyses were repeated using a competing risks model; (2) the main analyses were repeated using logistic regression; (3) participants in one-generation households were combined with two-generation households for reanalysis; (4) a more lenient cutoff for depressive symptoms (CES-D-10 score ≥ 18 and ≥10) was used as the outcome in a reanalysis.

Missing data were handled using multiple imputation. All *p* values were two-sided, with *p* < 0.05 considered statistically significant.

## 3. Results

### 3.1. Baseline Characteristics of Study Participants

A total of 7186 participants were included. The mean age was 55.84 years, and 47.3% were male. Participants in skipped-generation households were older, had lower educational attainment, and were more likely to reside in urban areas. Regarding health status, they reported lower self-rated health and accounted for the highest proportions of ADL and IADL impairments. In terms of lifestyle, individuals in skipped-generation households had the highest prevalence of insufficient sleep and were most likely to have two or more children. Table 1 presents detailed characteristics, and results based on non-imputed data (Table S1) were largely consistent with those shown in Table 1. For a detailed comparison of the characteristics of included and excluded participants, see Table S2. For details on missing values for covariates, see Table S3.

### 3.2. Association Between Baseline Family Structure and Incident Depressive Symptoms

**Figure 2:**
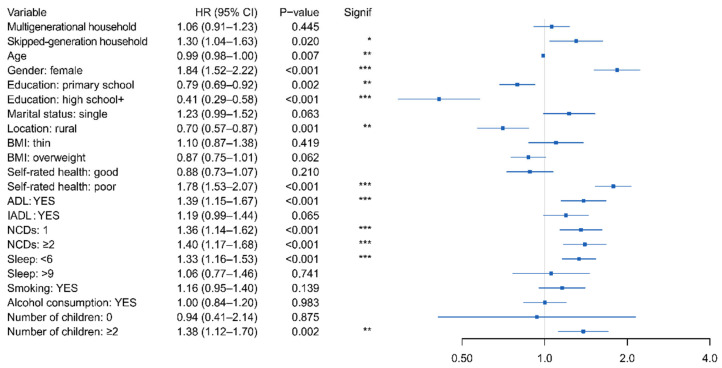
Association of baseline family structure with risks of depressive symptoms. * *p* < 0.05, ** *p* < 0.01, *** *p* < 0.001.

Supplementary Table S2 presents the association between baseline family structure and incident depressive symptoms. After adjustment for relevant covariates, participants in skipped-generation households showed a modest but statistically significant higher risk of developing depressive symptoms compared with those in two-generation households (HR = 1.30, 95% CI: 1.04–1.63); no significant difference was found among participants in multigenerational households. The results were illustrated in Figure 2.

### 3.3. Association Between Changes in Family Structure and Incident Depressive Symptoms

Supplementary Table S3 presents changes in family structure at the second follow-up. Among participants who were in two-generation households at baseline, 2017 transitioned to multigenerational households, and 650 transitioned to skipped-generation households; among skipped-generation household members at baseline, 13 transitioned to two-generation households, and 125 remained in skipped-generation households. Table 2 shows the association between changes in baseline household structure and incident depressive symptoms. Compared with participants who remained in stable two-generation households, those who transitioned from two-generation to skipped-generation households showed a significantly elevated risk of depressive symptoms (HR = 1.54, 95% CI: 1.06–2.23).

### 3.4. Subgroup Analysis of Family Structure and Incident Depressive Symptoms

**Figure 3:**
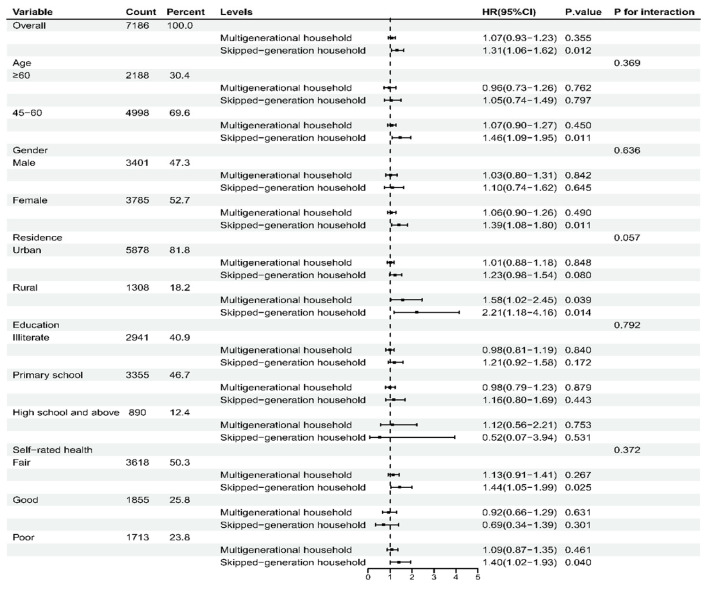
Subgroup of the association between family structure and incident depressive symptoms.

To explore the association between family structure and incident depressive symptoms across various sociodemographic factors, stratified by age, sex, residence, educational attainment, and self-rated health (Figure 3). The results consistently indicated that skipped-generation households were associated with an increased incidence of depressive symptoms across all subgroups. However, no significant interaction effects were observed (interaction *p* > 0.05).

### 3.5. Intergenerational Support as a Mediator of Family Structure and Depressive Symptoms

**Figure 4:**
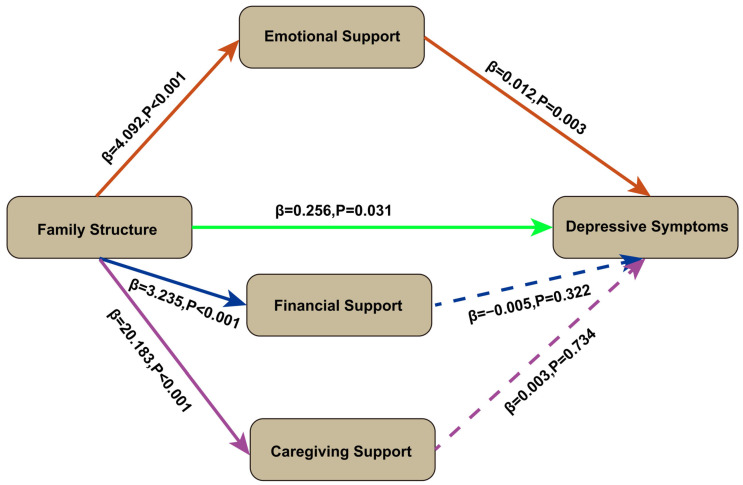
The mediating effect of intergenerational support.

The parallel mediation model was used to examine the mediating roles of three types of intergenerational support. The results showed that, compared with two-generation households, skipped-generation households had a significant direct effect on incident depressive symptoms (β = 0.3117, *p* = 0.0187). Bootstrap analysis indicated that the total indirect effect was statistically significant (Indirect Effect = 0.1453, 95% CI: 0.0163–0.2595). Among the specific mediation pathways, only emotional support showed a statistically significant indirect association (Indirect Effect = 0.1652, 95% CI: 0.0637–0.2671), whereas the indirect effects via financial support and caregiving support were not statistically significant (both confidence intervals included 0). These findings suggest that the association between skipped-generation households and incident depressive symptoms may be partially explained by emotional support, while no significant indirect associations were observed for financial support or caregiving support; see Figure 4.

### 3.6. Sensitivity Analyses

Sensitivity analyses were conducted to test robustness. Results from the competing risks model were consistent with the main analyses (Tables S6 and S7), and repeated analyses using logistic regression yielded similar conclusions (Tables S8 and S9). When one-generation households were combined with two-generation households, the association between baseline family structure and incident depressive symptoms remained stable (Table S10). Reanalysis using a more lenient depressive symptoms cutoff (CES-D-10 ≥ 18 and ≥10) produced consistent results (Table S11).

## 4. Discussion

Based on CHARLS data, this study systematically examined the correlation between family structure and its dynamic changes on incident depressive symptoms among middle-aged and older adults. Skipped-generation households were associated with a higher risk of depressive symptoms, while no association was observed for multi-generational households. Transitioning from a two-generation household to a skipped-generation household also elevated the likelihood of developing depressive symptoms, which highlights that the dynamic changes in family structure may have an important impact on the mental health of the elderly. Mediational analysis indicates that emotional support partially mediates the relationship between grandparent–grandchild household structure and depressive symptoms. For the baseline results, although we adjusted for a wide range of socio-demographic, health, and lifestyle covariates, we could not completely rule out residual confounding from unmeasured factors. At the same time, it should be emphasized that the above conclusion should not be interpreted as causality.

The risk of depressive symptoms in middle-aged and older adults is significantly higher in skipped-generation households compared with two-generation households, whereas no similar trend is observed for multigenerational households. Unlike two-generation households, skipped-generation households include grandchildren but no adult children [26]. This shift transforms grandparents from assisting caregivers into primary caregivers, a role change that may substantially increase both their daily and psychological burdens [27]. Grandparents assuming the main caregiving responsibilities often face greater time pressures, financial strain, and role conflicts, which can elevate levels of psychological distress [28,29]. In addition, long-term caregiving for grandchildren may limit older adults’ participation in social and leisure activities [30], exacerbating feelings of loneliness and helplessness. These observations align with the stress process model [31], which posits that role transitions expose individuals to external stressors that erode psychological resources and social support networks, increasing emotional exhaustion and stress responses, thereby raising the risk of depressive symptoms [32]. While these findings provide a plausible psychosocial explanation, the observed association should be interpreted with caution, given the potential for residual confounding and selection bias inherent in observational data. The elevated depressive symptoms risk in skipped-generation households may thus reflect the psychological burden experienced by middle-aged and older adults under the dual pressures of reduced social interaction and increased caregiving responsibilities [33].

When a family transitions from a two-generation to a skipped-generation household, the risk of depressive symptoms in middle-aged and older adults further increases. The observed transition from two-generation to skipped-generation households likely reflects broader intergenerational restructuring processes, including adult children leaving the household due to migration or independent living, changes in co-residence arrangements following family separation or bereavement, and the subsequent co-residence of grandchildren in the absence of their parents. These changes may shift older adults from a shared or supportive family role to primary caregivers for grandchildren. This transition often coincides with the birth or presence of grandchildren, which may lead adult children to redirect part of their emotional and material support from their parents to their own children. Existing studies suggest that adults with more children tend to provide less support to their parents, whereas most research indicates that intergenerational support has a protective effect on the mental health of middle-aged and older adults [34,35]. Consequently, when caregiving responsibilities increase while emotional and material support decline, older adults are more susceptible to depressive symptoms. In traditional Chinese culture, grandparental care is a family obligation [36]. More than half of Chinese grandparents report having participated in grandchild care [37], with approximately 41.43% in urban areas and 35.38% in rural areas [38]. However, related studies have also found that older adults who undertake grandchild caregiving commonly report higher levels of depressive symptoms [39,40].

Emotional support showed a significant indirect association in the relationship between skipped-generation households and depressive symptoms among middle-aged and older adults. In general, emotional support is widely recognized as a protective factor that may alleviate depressive symptoms and improve psychological well-being [41,42]. However, in our study, emotional support was unexpectedly associated with higher levels of depressive symptoms, which appears to differ from this conventional understanding. A more balanced interpretation is that the impact of emotional support may be context-dependent. While moderate emotional support may buffer psychological distress, high levels of support could inadvertently undermine older adults’ sense of autonomy and self-efficacy [43], potentially eliciting feelings of dependence, incompetence, or negative aging perceptions. In some cases, prolonged emotional involvement may also increase feelings of guilt or contribute to intergenerational tensions [44], which could adversely affect psychological well-being. At the same time, reverse causation should be considered. Older adults with emerging depressive symptoms or greater psychological vulnerability may receive increased emotional support from family members or actively seek such support as part of coping and help-seeking processes. This interpretation is supported by previous longitudinal evidence suggesting a bidirectional association between depressive symptoms and social support in older adults, whereby depressive symptoms may also influence subsequent levels of perceived or received support [45]. Therefore, the observed positive association in this study may partly reflect reverse causation or dynamic changes in support exchange patterns rather than a direct adverse effect of emotional support.

Subgroup analyses found that the association between skipped-generation households and depressive symptoms risk was stable across all stratified groups by age, gender, place of residence, educational attainment, and self-rated health. Middle-age and older adults in skipped-generation households within the younger age group (HR = 1.46) and rural areas (HR = 2.21) exhibited relatively higher depressive symptoms risk. This may relate to younger grandparents not yet being retired, thereby increasing their childcare burden in such households. In rural areas, caregiving was typically a long-term and primary caregiving arrangement [46], limited medical resources and reduced interaction with adult children may further contribute to the elevated risk of depressive symptoms [47]. Furthermore, sensitivity analysis results align with the primary findings, further validating the study’s reliability. However, the formal interaction test of all subgroup factors did not reach significance (all interactions *p* > 0.05). Therefore, these subgroup differences should be regarded as exploratory results and cannot be used as evidence of effect modification.

Overall, these findings underscore the importance of considering both family structure and its dynamic transitions in understanding depressive symptoms among middle-aged and older adults. By showing that emotional support is associated with the relationship between skipped-generation living arrangements and incident depressive symptoms, our findings are consistent with a pattern of partial mediation, although causal mediation cannot be inferred. These results contribute to the psychiatric epidemiology literature by extending existing evidence on social determinants of depressive symptoms to a dynamic, family-based framework and suggest that social role transitions may represent a potential pathway through which family contexts are linked to mental health trajectories in aging populations.

Despite the strengths of a large sample size and longitudinal design, several limitations should be noted. First, data on intergenerational family relationships and depressive symptoms were based on participant self-reports, which may introduce information bias. Secondly, data from CHARLS surveys may pose a risk of selection bias due to lack of response, sample loss, and other factors. Thirdly, the sample is limited to middle-aged and elderly people in China, and cannot be extended to Chinese middle-aged and elderly people who live alone or only with their spouses. Fourthly, depressive symptoms were assessed using the CES-D-10 screening tool, and the conservative cutoff (≥20) may have underestimated the incidence of depressive symptoms. Fifth, the measurement criteria for intergenerational support in this study are relatively limited and may not fully capture important dimensions of support, including relationship quality, directionality, adequacy, reciprocity, or perceived burden, all of which may affect mental health outcomes. Sixth, the significance of family changes is uncertain because CHARLS did not record the reasons for the changes. Seventh, the association between excessive emotional support and increased risk of depressive symptoms in older adults is speculative and requires further verification. Finally, although this study found an association between intergenerational families and the risk of depressive symptoms, as well as significant indirect associations involving emotional support, the observational nature of the study excluded causal inferences about mediating pathways. It cannot be ruled out that a reverse causal relationship and residual confounding of unmeasured factors (such as income and psychosocial resources) may still exist.

## 5. Conclusions

This study found that depressive symptoms among middle-aged and older adults were associated with intergenerational family structure and its transitions. Emotional support may play a mediating role in this association, suggesting that the impact of family structure on mental health extends beyond changes in caregiving burden to include dynamic adjustments in emotional relationships and social support. These findings highlight the importance of supporting the psychological adaptation of older adults during changes in intergenerational family arrangements. However, this study did not establish causal relationships or fully elucidate the underlying mechanisms.

## Figures and Tables

**Table 1:** Baseline characteristics of study participants by family structure.

Variables	Category	Overall	Two-Generation Household	Multigenerational Household	Skipped-Generation Household	*p*
Number (%)		7186	3148 (43.8)	3354 (46.7)	684 (9.5)	
Age, mean (SD), years		55.84 (8.4)	52.04 (7.2)	58.43 (8.2)	60.60 (7.4)	<0.001
Gender, n (%)	Male	3401 (47.3)	1532 (48.7)	1543 (46.0)	326 (47.7)	0.098
	Female	3785 (52.7)	1616 (51.3)	1811 (54.0)	358 (52.3)	
Education, n (%)	Illiterate	2941 (40.9)	937 (29.8)	1626 (48.5)	378 (55.3)	<0.001
	Primary school	3355 (46.7)	1672 (53.1)	1428 (42.6)	255 (37.3)	
	High school or above	890 (12.4)	539 (17.1)	300 (8.9)	51 (7.5)	
Marital status, n (%)	Married	6583 (91.6)	2973 (94.4)	2981 (88.9)	629 (92.0)	<0.001
	Single	603 (8.4)	175 (5.6)	373 (11.1)	55 (8.0)	
Residence, n (%)	Rural	5878 (81.8)	2494 (79.2)	2805 (83.6)	579 (84.6)	<0.001
	Urban	1308 (18.2)	654 (20.8)	549 (16.4)	105 (15.4)	
Number of children, n (%)	One-child family	1262 (17.6)	714 (22.7)	487 (14.5)	61 (8.9)	<0.001
	No children	60 (0.8)	28 (0.9)	6 (0.2)	26 (3.8)	
	Multi-child family	5864 (81.6)	2406 (76.4)	2861 (85.3)	597 (87.3)	
BMI, n (%)	Normal	3762 (52.4)	1584 (50.3)	1786 (53.2)	392 (57.3)	<0.001
	Thinness	549 (7.6)	166 (5.3)	297 (8.9)	86 (12.6)	
	Overweight	2875 (40.0)	1398 (44.4)	1271 (37.9)	206 (30.1)	
Self-rated health, n (%)	Fair	3618 (50.3)	1574 (50.0)	1683 (50.2)	361 (52.8)	0.001
	Good	1855 (25.8)	868 (27.6)	848 (25.3)	139 (20.3)	
	Poor	1713 (23.8)	706 (22.4)	823 (24.5)	184 (26.9)	
ADL, n (%)	No	6404 (89.1)	2863 (90.9)	2966 (88.4)	575 (84.1)	<0.001
	Yes	782 (10.9)	285 (9.1)	388 (11.6)	109 (15.9)	
IADL, n (%)	No	6343 (88.3)	2859 (90.8)	2898 (86.4)	586 (85.7)	<0.001
	Yes	843 (11.7)	289 (9.2)	456 (13.6)	98 (14.3)	
NCDs, n (%)	0	2638 (36.7)	1286 (40.9)	1136 (33.9)	216 (31.6)	<0.001
	1	2200 (30.6)	952 (30.2)	1053 (31.4)	195 (28.5)	
	≥2	2348 (32.7)	910 (28.9)	1165 (34.7)	273 (39.9)	
Sleep hours, n (%)	6~9	5056 (70.4)	2310 (73.4)	2284 (68.1)	462 (67.5)	<0.001
	<6	1820 (25.3)	701 (22.3)	922 (27.5)	197 (28.8)	
	>9	310 (4.3)	137 (4.4)	148 (4.4)	25 (3.7)	
Smoking, n (%)	No	5022 (69.9)	2165 (68.8)	2384 (71.1)	473 (69.2)	0.117
	Yes	2164 (30.1)	983 (31.2)	970 (28.9)	211 (30.8)	
Alcohol consumption, n (%)	No	5585 (77.7)	2443 (77.6)	2621 (78.1)	521 (76.2)	0.516
	Yes	1601 (22.3)	705 (22.4)	733 (21.9)	163 (23.8)	

SD, standard deviation; BMI, body mass index; ADL, activities of daily living; IADL, instrumental activities of daily living; NCDs, non-communicable diseases.

**Table 2:** Association of changes in family structure with risks of incident depressive symptoms.

Changes	HR1 ^a^ (95% CI)	P1 ^a^	HR2 ^b^ (95% CI)	P2 ^b^
Stable Two-generation household	1 (reference)		1 (reference)	
Two-generation to Multigenerational	1.32 (0.95–1.83)	0.102	1.37 (0.98–1.90)	0.061
Two-generation to Skipped-generation	1.50 (1.03–2.17)	0.032	1.54 (1.06–2.23)	0.023
Stable Multigenerational household	1 (reference)		1 (reference)	
Multigenerational to Two-generation	0.81 (0.48–1.36)	0.433	0.85 (0.51–1.43)	0.545
Multigenerational to Skipped-generation	1.06 (0.85–1.31)	0.568	1.06 (0.84–1.33)	0.633
Stable Skipped-generation household	1 (reference)		1 (reference)	
Skipped-generation to Two-generation	0.84 (0.52–1.39)	0.514	0.89 (0.54–1.46)	0.640
Skipped-generation to Multigenerational	0.43 (0.06–3.11)	0.406	0.45 (0.06–3.25)	0.430

^a^ HR1 and P1 were unadjusted. ^b^ HR2 and P2 were adjusted for age, gender, education, marital status, residence, BMI, ADL, IADL, NCDs, sleep hours, smoking, alcohol consumption, and number of children.

## Data Availability

The CHARLS data are available at http://charls.pku.edu.cn/ (accessed on 9 October 2025).

## Supplementary Materials

The following supporting information can be downloaded at: https://www.mdpi.com/article/10.3390/healthcare14131855/s1, Table S1. Baseline Characteristics of Participants by Family Structure Using Non-imputed Data. Table S2. Comparison of Baseline Characteristics Between Included and Excluded Participants. Table S3. Missing Data for Covariates. Table S4. Association of Baseline Family Structure with Risks of Depressive Symptoms. Table S5. Number and Percentage of the Changes in Family Structure. Table S6. Association of Baseline Family Structure with Depressive Symptoms Using a Competing Risk Model. Table S7. Association of Changes in Family Structure with Incident Depressive Symptoms Using a Competing Risk Model. Table S8. Association of Baseline Family Structure with Depressive Symptoms Using Logistic Regression. Table S9. Association of Changes in Family Structure with Incident Depressive Symptoms Using Logistic Regression. Table S10. Association of Baseline Family Structure with Risks of Depressive Symptoms (Merge One-generation Household). Table S11. Association of Baseline Family Structure with Risks of Depressive Symptoms (Change the outcome definition).
